# CT-based visualization of aortic valve morphology: from 3D energy-integrating CT to 4D photon counting CT

**DOI:** 10.3389/fcvm.2025.1721746

**Published:** 2026-01-12

**Authors:** Alison M. Pouch, Jessie N. Dong, Harold I. Litt, Brittany J. Cannon, Melanie Freas, Victor Ferrari, Thomas G. Gleason, Matthew A. Jolley, Natalie Yushkevich, Jessica Nunez, Jilei Hao, Zaiyang Guo, Shir Goldfinger, John Kelly, Joseph E. Bavaria, Nimesh D. Desai

**Affiliations:** 1 Department of Radiology, University of Pennsylvania, Philadelphia, PA, United States; 2 Department of Bioengineering, University of Pennsylvania, Philadelphia, PA, United States; 3 Division of Cardiovascular Surgery, Department of Surgery, University of Pennsylvania, Philadelphia, PA, United States; 4 Division of Cardiovascular Medicine, Department of Medicine, University of Pennsylvania, Philadelphia, PA, United States; 5 Asheville Heart, Asheville, NC, United States; 6 Division of Cardiothoracic Anesthesiology, Department of Anesthesiology and Critical Care Medicine, Children's Hospital of Philadelphia, Philadelphia, PA, United States; 7 Department of Cardiac Surgery, Thomas Jefferson University, Philadelphia, PA, United States

**Keywords:** aortic valve (bicuspid), 4D computed tomography, photon counting computed tomography, valve repair, surgical planning

## Abstract

**Background:**

While 4D contrast-enhanced computed tomography (CT) is used to plan cardiovascular interventions such as transcatheter valve replacement, it is not yet routinely used to characterize minimally calcified aortic valves for planning of surgical valve repair. It is widely recognized that aortic valve morphology has implications for the durability of valve repair surgery.

**Purpose:**

The objective is to demonstrate the potential of CT image segmentation for elucidating aortic valve morphology prior to surgery and to illustrate a potential benefit of 4D CT and photon counting CT (PCCT) for patient-specific modeling of dysmorphic aortic valves.

**Materials and methods:**

This observational series includes nine patients who were suspected to have minimally calcified bicuspid aortic valve morphology on transthoracic echocardiography (TTE). Mean age was 53 +/- 13 years and seven patients were male. For the seven patients who underwent aortic root surgery, CT-based segmentation of the aortic valve was compared to echocardiographic interpretation and direct intraoperative visualization of valve morphology. Two patients who have not yet undergone aortic surgery were imaged longitudinally with 4D energy-integrating detector CT (EID-CT) and 4D PCCT, and the morphological interpretation of the aortic valve was compared to previous TTE reports.

**Results:**

In most surgical cases, CT-based segmentation and direct visualization of the valve revealed morphological features not previously confirmed on TTE, particularly related to the cusp fusion pattern. Moreover, 4D CT enabled morphological assessment at both systole and diastole, which captured maximal cusp separation and valve closure. PCCT images were reconstructed with slice thickness as low as 0.2 mm, and revealed detailed dysmorphic features such as a small accessory cusp with fistula and a double raphe in separate patients.

**Conclusion:**

4D CT-based segmentation has the potential to dynamically capture aortic valve features that are relevant to risk stratification and surgical planning at high spatial resolution.

## Introduction

4D (3D + time) contrast-enhanced computed tomography (CT) has become routine for planning cardiovascular interventions such as transcatheter aortic valve replacement (1) and assessing bioprosthetic valve thrombosis (2), with recent studies demonstrating added value of photon counting CT (PCCT) (3–5). Multi-phase CT reconstruction has demonstrated promise (6–9), but is not routinely used to assess minimally calcified, regurgitant valves – particularly bicuspid aortic valves (BAVs) – for risk stratification and planning valve repair. In this context, transthoracic echocardiography (TTE) is the first-line diagnostic and phenotyping modality. In the operating room, transesophageal echocardiography (TEE) is acquired while the patient is sedated to obtain higher quality views of valve morphology and function to inform surgical decision making. Although echocardiography does not use ionizing radiation, TTE has limited acoustic windows that make valve morphology difficult to discern, and TEE is generally acquired when the patient is already anesthetized in the operating room. Thus, surgical planning is often carried out quickly in the operating room, and intraoperative “discoveries” on TEE and direct visualization of the aortic valve while the heart is arrested during cardiopulmonary bypass can substantially impact the surgical plan (10).

Accurate discrimination of aortic valve morphology is critical to risk stratification for surgical repair vs. replacement of regurgitant valves. Trileaflet and symmetrical BAVs (i.e., valves with a 180–180 cusp orientation) are generally more conducive to repair than unicuspid, highly asymmetric bicuspid, or quadricuspid aortic valves (11). Additional characteristics, such as size of the non-fused cusp and extent of calcification, influence valve repair durability (12). This has motivated development of morphological classification schemes – such as those proposed by Sievers et al. (13), De Kerchove et al. (14), and Michelena et al. (15) – which have been adopted for image-based aortic valve interpretation.

Given the importance of aortic valve morphology and the challenges of its interpretation on routine TTE, CT-based aortic valve segmentation may be a complementary tool to echocardiography and direct intra-operative visualization when planning surgical intervention for aortic regurgitation. 4D CT and PCCT may be particularly beneficial for visualizing aortic valve dynamics at high spatial resolution. The purpose of this study is (1) to demonstrate retrospectively the potential for CT-based segmentation to elucidate aortic valve morphology before entering the operating room and (2) to qualitatively illustrate the performance of 4D CT and PCCT for detailed assessment of aortic valve morphology relative to intraoperative inspection.

## Materials and methods

### Research protocols and patient inclusion

**Figure 1:**
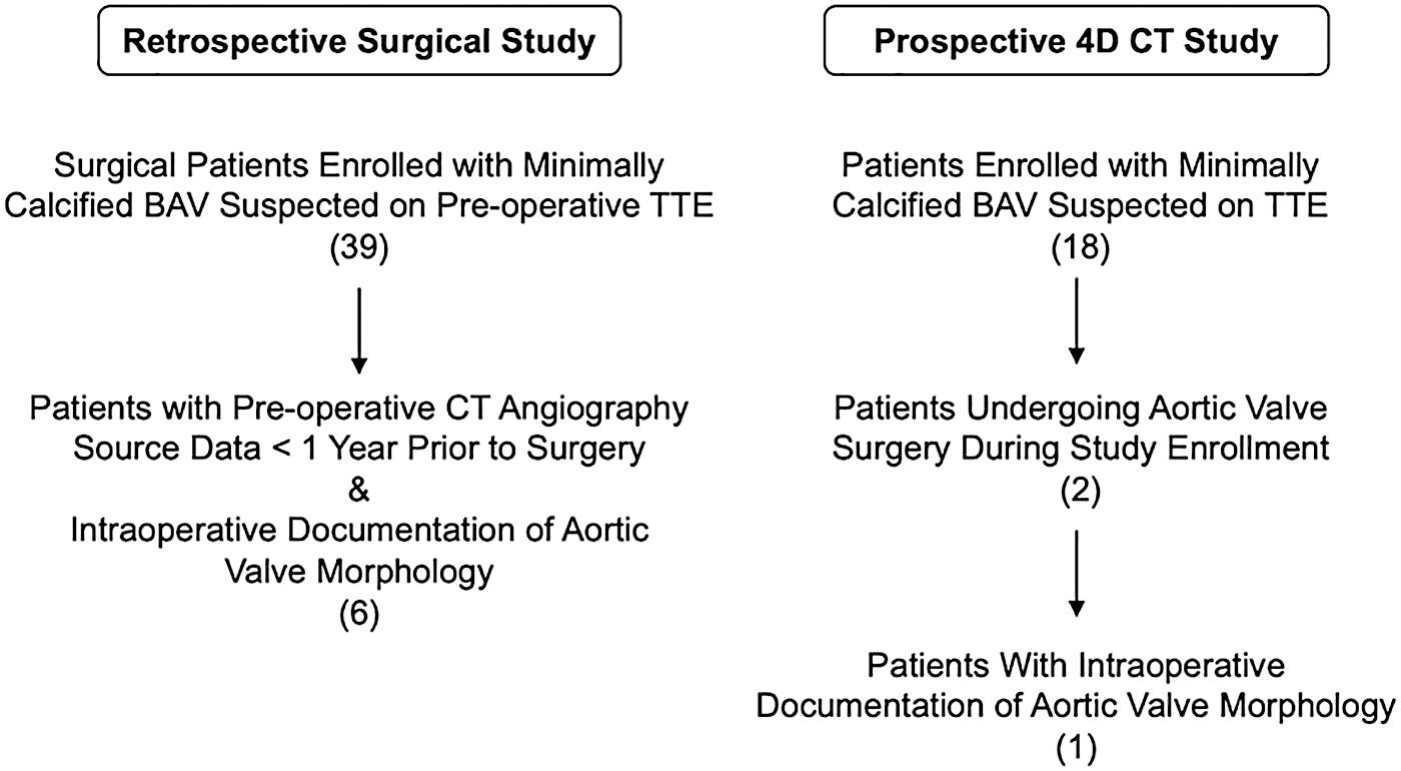
Patient inclusion from two research protocols.

CT images of minimally calcified aortic valves were obtained from two research protocols conducted between December 2020 and November 2023: a retrospective study of routine clinical BAV imaging and a prospective 4D CT observational study of BAVs (Figure 1). In both studies, the eligibility criteria included adults with documentation of a BAV on clinical imaging and any degree of aortic regurgitation. The exclusion criteria were prior aortic valve surgery and/or aortic valve calcification greater than mild on clinical imaging. Both protocols were approved by the Institutional Review Board at the University of Pennsylvania and recruit adult patients who are suspected to have a BAV with minimal or no calcification based on TTE. From these protocols, a total of seven cases contained the following information: at least one pre-operative 2D TTE report with aortic valve assessment, pre-operative contrast-enhanced CT angiography of the chest or cardiac CT images, a TEE-based assessment of aortic regurgitation in the operating room, and intraoperative documentation of aortic valve morphology via direct visual inspection while the patient was on cardiopulmonary bypass. Two additional cases were curated from the prospective research study with PCCT imaging. In total, there were nine patient cases curated for this study, the first seven of whom had direct visual confirmation of aortic valve morphology. Two of the nine datasets have been used in a previous study (16). This prior article used manual valve segmentations to train a deep learning model for fully automatic aortic valve segmentation, whereas this manuscript focuses on the morphological findings of manual segmentation and compares imaging with energy-integrating detector CT (EID-CT) and PCCT.

### Patient and imaging characteristics

Patient characteristics are summarized in Table 1. The majority (78%) were male, given the known male predominance of BAV disease (17). Age ranged from 25 to 65 years. The first seven patients had suspected BAV on routine 2D TTE and underwent CT imaging and surgery on the aortic root, valve, and/or the ascending aorta/arch. Six of these had clinically performed EID-CT angiography of the chest using a prospectively ECG-triggered high pitch helical or axial acquisition resulting in images at a single cardiac phase (typically late diastolic), and one had retrospectively ECG-gated 4D EID-CT of the aortic valve for research with 20 phases reconstructed over the cardiac cycle (Table 2). The latter scan was acquired with a Somatom Force CT scanner (Siemens Healthineers, Malvern, PA). The final two patients are under clinical surveillance and have not undergone aortic valve surgery but had suspected BAV on TTE and underwent a retrospectively ECG-gated 4D research CT of the aortic valve with the EID-CT scanner at baseline and a PCCT scanner (Naeotom Alpha, Siemens Healthineers) one year later. Overall, the CT images had in-plane resolution ranging from 0.32 to 0.89 mm and slice thickness from 0.2 to 2 mm. Iodinated contrast was administered at a rate and volume that depended upon scan duration and kVp, but was typically 60–100 mL of iopamidol 370 mg I/mL (Isovue 370, Bracco Diagnostics, Monroe Township, NJ) at 3–5 mL/s. ECG-based tube current modulation was not used during retrospective gating in this pilot project.

**Table 1:** Patient characteristics.

Case	Sex	Age (yrs)	BMI (kg/m^2^)	LVEF (%)	Primary indication for surgery	Aortic intervention performed
1	M	54	27.0	60	Ascending/aortic arch aneurysm, mild AR	Transverse aortic arch graft; ascending aortic aneurysm repair; aortic valve repair
2	F	57	33.2	58	Ascending/aortic arch aneurysm	Transverse aortic arch graft; ascending aortic aneurysm repair; limited aortic valve repair
3	M	59	26.9	58	Ascending/aortic arch aneurysm	Aortic arch graft; valve sparing aortic root reconstruction
4	M	50	32.9	45	Severe AR	Aortic root reimplantation valve sparing procedure; aortic valve repair
5	M	25	24.7	55	Aortic root aneurysm, severe AR	Aortic root reimplantation valve sparing surgery; aortic valve repair
6	M	65	26.7	55	Ascending/aortic arch aneurysm, moderate AR	Transverse aortic arch graft; aortic root reimplantation valve sparing surgery
7	M	59	32.4	60	Ascending/aortic arch aneurysm, severe AR	Transverse aortic arch graft; Bio-Bentall procedure
8	F	65	28.8	68	None (surveillance)	–
9	M	41	32.5	58	None (surveillance)	–

BMI, body mass index; LVEF, left ventricular ejection fraction; AR, aortic regurgitation.

**Table 2:** Summary of the aortic valve TTE classification, CT image characteristics and model visualization, and direct intraoperative inspection of the valve.

Case	TTE classification	CT characteristics	CT model visualization	Intraoperative inspection
1	BAV Type 1 L/R fusion	Diastolic EID-CT In plane: 0.45 × 0.45 mm Slice thickness: 0.75 mm Slice interval: 0.4 mm	BAV Type 1 L/R fusion	BAV Type 1 L/R fusion
2	BAV Type 1 L/R fusion	Diastolic EID-CT In plane: 0.39 × 0.39 mm Slice thickness: 0.75 mm Slice interval: 0.4 mm	BAV Type 1 L/R fusion	BAV Type 1 L/R fusion
3	BAV Type 1 R/N fusion	Diastolic EID-CT In plane: 0.85 × 0.85 mm Slice thickness: 1.0 mm Slice interval: 1.0 mm	BAV Type 0 Symmetric	BAV Type 0 Symmetric
4	BAV with variable interpretations (Type 1 R/N fusion, Type 1 L/N fusion)	Systolic EID-CT In plane: 0.89 × 0.89 mm Slice thickness: 1.0 mm Slice interval: 1.0 mm	BAV Type 0 Symmetric	BAV Nearly symmetric, very small L/R raphe
5	BAV without morphological specification	Diastolic EID-CT In plane: 0.65 × 0.65 mm Slice thickness: 2.0 mm Slice interval: 1.2 mm	BAV Type 1 R/N fusion	BAV Type 1 R/N fusion
6	Variable interpretations (TAV, possible BAV)	Diastolic EID-CT In plane: 0.48 × 0.48 mm Slice thickness: 0.75 mm Slice interval: 0.4 mm	TAV	TAV Asymmetrically sized cusps
7	Variable interpretations (TAV, possible BAV)	4D EID-CT In plane: 0.38 × 0.38 mm Slice thickness: 0.5 mm Slice interval: 0.3 mm	TAV Calcifications on each cusp	TAV Calcifications on each cusp
8	Variable interpretations (TAV, BAV, “abnormal”)	4D PCCT In plane: 0.43 × 0.43 mm Slice thickness: 0.2 mm Slice interval: 0.2 mm	Dysmorphic AV Small accessory cusp with fistula at L/R commissure	–
9	BAV Type 1 L/R fusion	4D PCCT In plane: 0.32 × 0.32 mm Slice thickness: 0.2 mm Slice interval: 0.2 mm	Unicuspid AV Type 2 L/R-R/N fusion	–

The Sievers classification scheme is used to describe aortic valve morphology.

### CT-Based aortic valve modeling

CT images were exported in DICOM format and de-identified, and aortic valve segmentation carried out and reviewed by valve modelers blinded to the surgical findings. The aortic root and cusps were manually traced in 3D using ITK-SNAP (18) and smoothed. For the single-phase scans, one 3D model of the aortic valve apparatus was generated. For the 4D scans, the valve was segmented in two 3D image volumes (one systolic and one diastolic), and the segmentations propagated to the other 3D volumes using deformable image registration as described by Aggarwal et al. (19). The result was a model of aortic valve morphology and motion over one complete cardiac cycle, consisting of 20 CT image volumes.

For each case, the 2D TTE interpretation and the 3D CT digital model visualization were compared and verified against intraoperative footage and the surgeon's interpretation of the exposed valve, when available.

## Results

**Figure 2:**
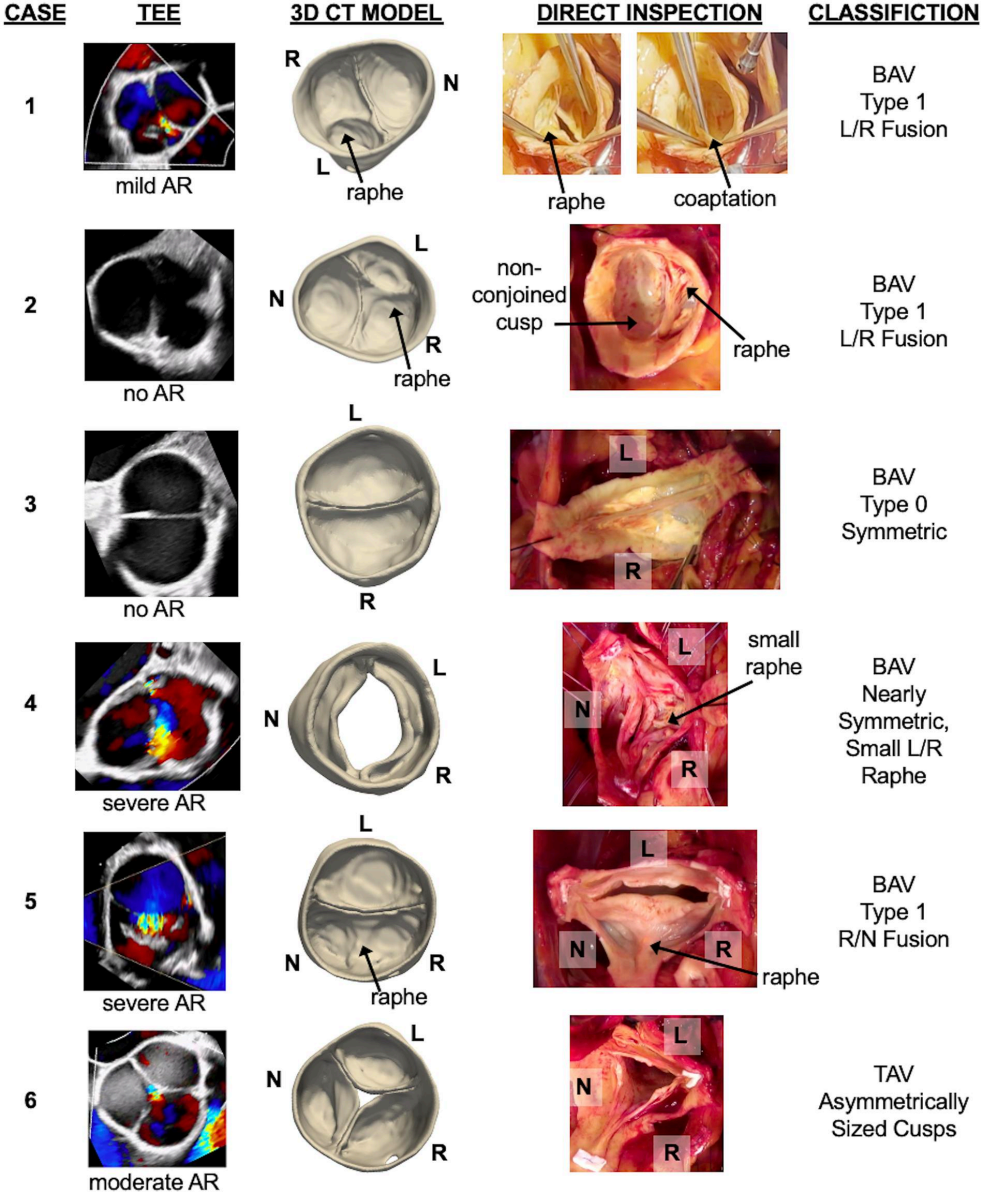
Six cases demonstrating pre-operative CT segmentation of the aortic valve relative to direct intraoperative visualization of the valve. Each case includes the intraoperative transesophageal echocardiogram (TEE) and the degree of aortic regurgitation (AR), an anatomical model of the aortic valve derived from single-phase CT, direct intraoperative inspection of the aortic valve, and the surgeon's classification of aortic valve morphology based on direct visualization. L, R, and N annotate the orientation of the left, right, and non-coronary cusps, respectively.

**Figure 3:**
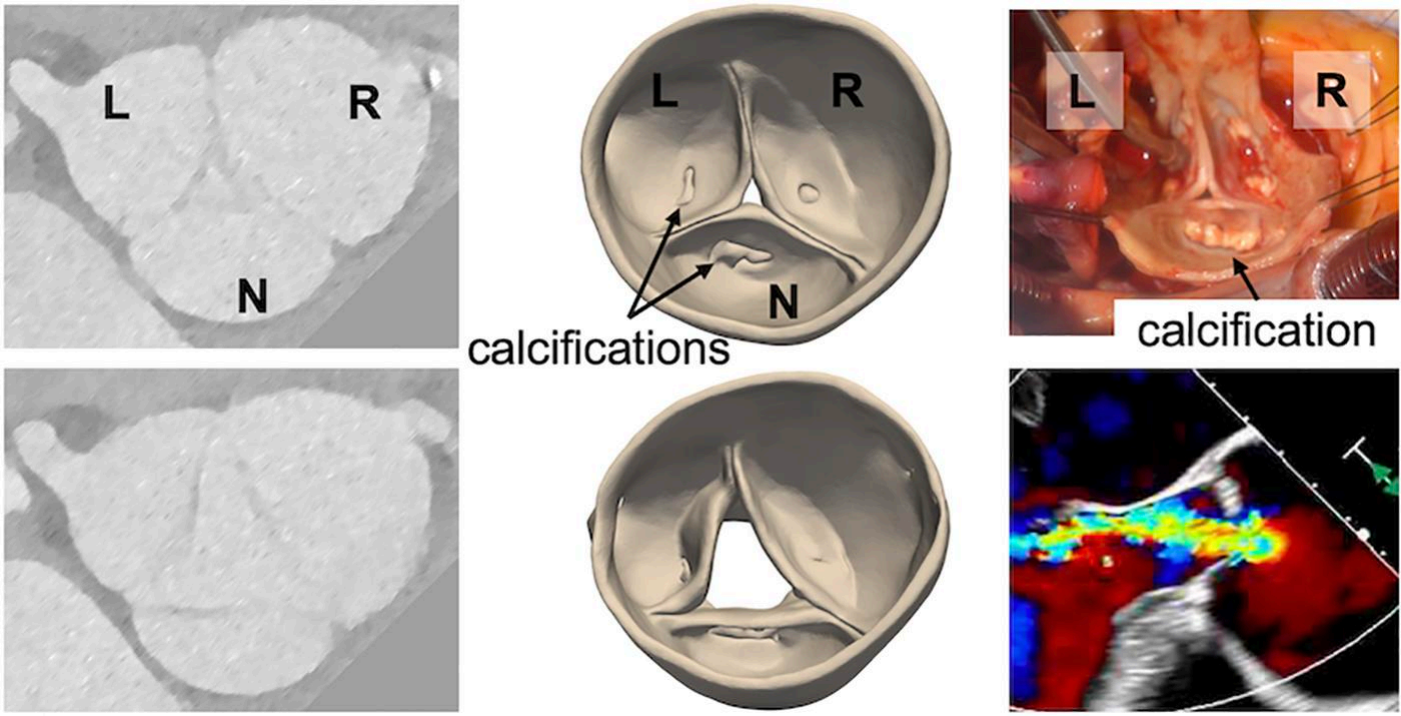
A severely regurgitant aortic valve that was suspected to be bicuspid but observed to be trileaflet on direct intraoperative inspection. (Left) Grayscale images from the pre-operative CT, (center) 3D models of the aortic valve obtained by CT segmentation, (right) direct visualization of the aortic valve and pre-operative transesophageal echocardiogram showing the regurgitant jet.

**Figure 4:**
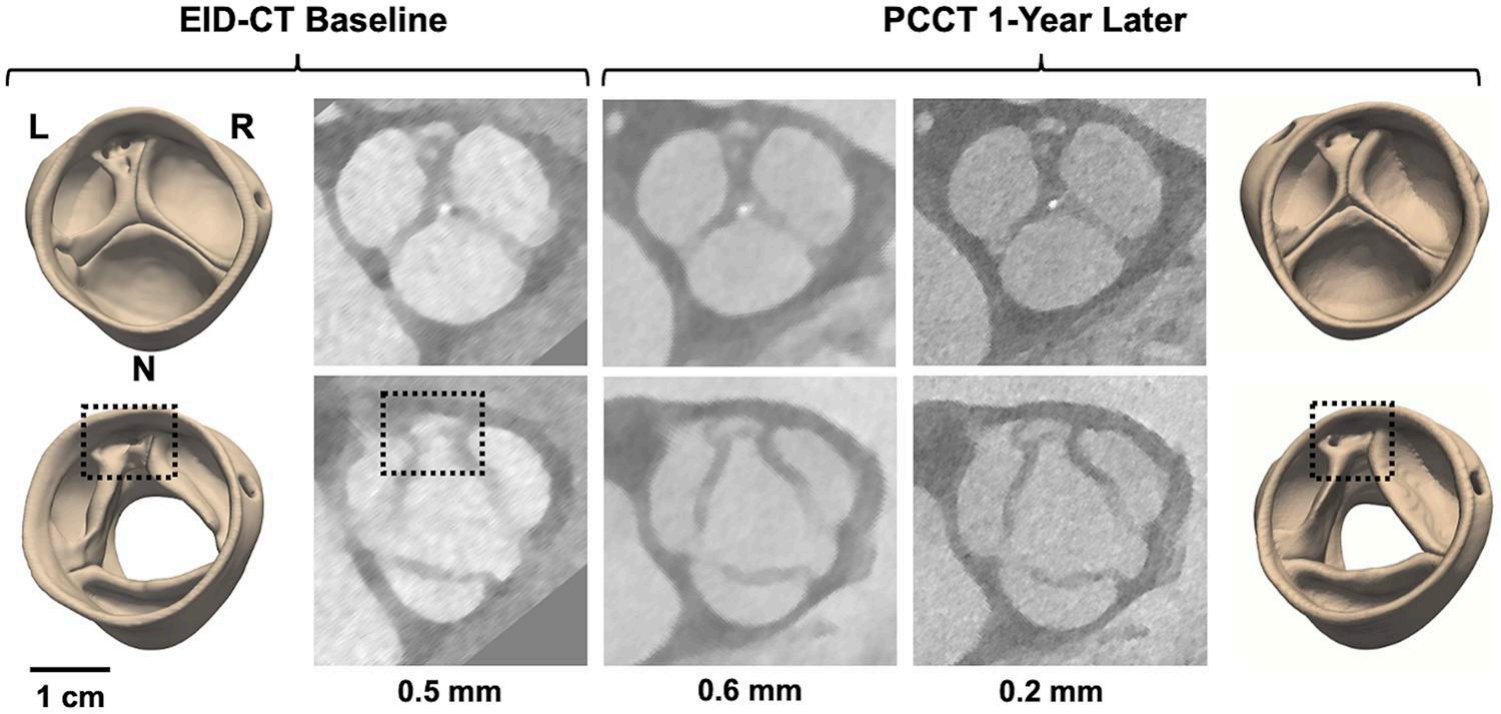
4D CT modeling of a potentially dysmorphic aortic valve imaged with EID-CT at baseline and PCCT one year later. The top row illustrates image reconstructions and segmentations at diastole, the bottom row at systole. CT images were reconstructed with various slice thicknesses: 0.5 mm for EID-CT and 0.6 and 0.2 mm for PCCT. L, R, and N annotate the orientation of the left, right, and non-coronary cusps, respectively. The dashed box annotates the location of an accessory cusp with fistula observed near the L/R commissure.

**Figure 5:**
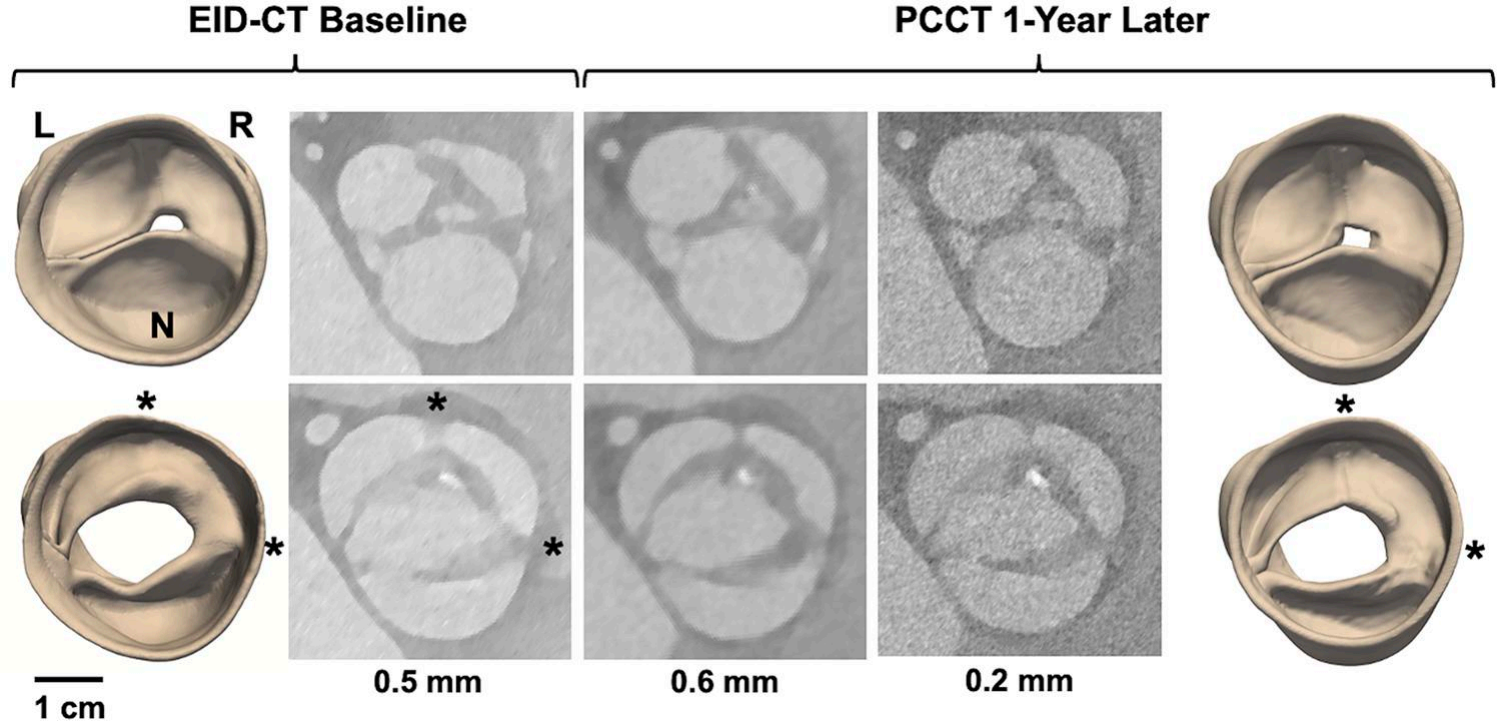
4D CT modeling of a potentially unicuspid aortic valve imaged with EID-CT at baseline and PCCT one year later. The top row illustrates image reconstructions and segmentations at diastole, the bottom row at systole. CT images were reconstructed with various slice thicknesses: 0.5 mm for EID-CT and 0.6 and 0.2 mm for PCCT. L, R, and N annotate the orientation of the left, right, and non-coronary cusps, respectively. The asterisks annotate cusp fusions observed at the L/R and R/N commissures.

Imaging characteristics of the nine cases are summarized in Table 2, including routine pre-operative 2D TTE interpretation, CT image characteristics, and findings of the intraoperative valve inspection. The 2D TEE Doppler images, CT-derived aortic valve models, and direct intraoperative valve inspections are shown side-by-side in Figure 2. The results of 4D CT are illustrated in Figure 3 (EID CT) and in Figures 4, 5 (PCCT).

Cases 1 and 2 had the most common BAV phenotype: fusion of the left and right coronary cusps with a single raphe (Sievers Type 1). The morphological classification on routine 2D TTE was consistent with the CT-derived model and direct visualization of the valve in both cases. Additional detail can be appreciated in the CT-derived models: the raphe of both valves divided the conjoined cusp asymmetrically, with the right coronary cusp dominant in size over the left coronary cusp. The origin of mild aortic regurgitation is not immediately apparent from the diastolic CT model from Case 1; the intraprocedural short-axis TEE with Doppler indicates a coaptation defect near the left/non-coronary commissure. Case 2 had no aortic regurgitation at the time of surgery and the cusps appear to fully coapt in the CT-derived model.

Cases 3 and 4 had a less common BAV phenotype with two symmetrical leaflets. For Case 3, the direct intraoperative visualization confirmed a Sievers Type 0 configuration. TTE interpretation misclassified the valve as Sievers Type 1 R/N fusion, whereas the CT-derived model demonstrates Sievers Type 0 with lateral cusp orientation and one coronary ostium per sinus. For Case 4, there were mixed morphological interpretations on separate TTEs and the CT-derived model demonstrates a symmetrical aortic valve. The intraoperative visualization confirmed near-perfect symmetry but with a very small L/R raphe, which was not apparent in the CT model. The coaptation defect could not be assessed from CT since the images were obtained during systole.

Cases 5 and 6 were suspected to have abnormal valve morphology on TTE, but a detailed classification was not provided. For Case 5, the CT model showed a Sievers Type 1 R/N fusion, consistent with the intraoperative visualization. Color Doppler TEE suggested a central coaptation defect, which was not apparent in the CT model. Case 6 revealed a trileaflet valve on both CT modeling and intraoperative inspection. Both demonstrate an asymmetrically larger right coronary cusp, and the CT model further captures a central coaptation that is consistent with the TEE assessment.

Case 7 involved 4D CT acquisition from a patient who had serial TTEs with variable interpretations of aortic valve morphology, including both trileaflet and bicuspid classifications. Upon direct visualization in the operating room, the valve was observed to have three distinct cusps with moderate calcification at the base of each cusp. The systolic CT model likewise revealed a trileaflet valve with an asymmetric aortic root aneurysm and calcifications on each cusp. The diastolic CT model revealed a central coaptation defect, consistent with the intraoperative TEE exam (Figure 3). Although the valve morphology and cusp sizes were favorable for valve repair, surgical replacement was performed due to the extent of cusp calcification.

Cases 8 and 9 are patients under clinical surveillance who underwent both 4D EID-CT and PCCT. Both had TTE reports of a BAV; however, more complex morphological details are observed in the 4D CT data. In Case 8, systolic CT reveals three distinct cusps with a dysmorphism – potentially a small accessory cusp with fistula – between the left and right coronary cusps. This defect is likewise visible in the diastolic reconstruction and is apparent in both the 3D models and source images (Figure 4). Case 9 was classified as Sievers Type 1 L/R fusion on TTE, but appears to have an additional raphe between the right and non-coronary cusps on high-resolution CT. This suggests it may be a unicuspid variant with two raphes and a single L/N commissure (Sievers Type 2 L/R-R/N) (Figure 5).

Image resolution varied among the datasets, with the 4D scans having slice thicknesses of 0.2–0.5 mm and single-phase CT having slice thicknesses of 0.75–2 mm. The interpretation of Case 4 suggests that CT spatial resolution close to 1.0 mm may not be adequate to capture details such as small raphes. However, this resolution was adequate to characterize cusp symmetry in Case 3, which did not have any finely detailed dysmorphic features on the intraoperative exam. Regurgitant orifices were not visible in Case 1 and 5 diastolic reconstructions, which may be related to limited spatial resolution and/or inability to assess dynamics of cusp closure on static CT. However, CT and TEE were not acquired simultaneously, which precludes direct comparison of valvular regurgitation.

## Discussion

This study demonstrates the value of contrast-enhanced CT, particularly 4D CT, in elucidating aortic valve morphology and cusp symmetry in non-calcific valvular disease. Of the 7 aortic valves with direct visual confirmation of morphology, 4 cases had either morphological misclassification or inconclusive interpretation on routine TTE. The 3D CT-derived models, on the other hand, enabled visualization of valve symmetry and morphology with the exception of detail missed in a lower-resolution dataset (small raphe of Case 4) and lack of a visible regurgitant orifice in Cases 1 and 5. Given the difficulty of obtaining acoustic windows with routine 2D TTE, CT provides an opportunity for highly detailed morphological interpretation in advance of the patient undergoing TEE.

The results point to added benefit of 4D CT over single-phase imaging. Aortic valve assessment at systole makes cusp fusion easier to discern since the cusps are maximally separated, while assessment at diastole potentiates identification of coaptation defects. Thus, 4D imaging enables the complementary benefits of multi-phase interpretation and allows the radiologist to investigate whether anomalies (such as the dysmorphism of Case 8) are consistent across the cardiac cycle, rather than an image artifact at a single phase. When a regurgitant orifice is not visible in a diastolic CT reconstruction, correlation with Doppler echocardiography (as in Cases 1 and 5) can facilitate localization of the coaptation defect.

Building on previous work that demonstrates differentiation of aortic valve morphology in CT source images (20), our findings also point to the value of *segmenting* the aortic valve apparatus. Conventionally, CT is visualized in 2D axial or reformatted cross-sections. However, it can be challenging to interpret complex defects in a slice-by-slice visualization. For example, the CT volumes of Cases 8 and 9 must be sliced along specific axes to capture the detailed dysmorphic features in a cross-sectional display, but those features are readily apparent in the 3D digital models (Figures 4, 5). Moreover, Case 7 was likely misclassified as a BAV on TTE since the asymmetric root aneurysm stretched the R/N commissure, which can be observed in the 3D digital model (Figure 3) but may be mischaracterized as cusp fusion on cross-sectional visualization. It is notable that the valve segmentations presented in this work were generated with open-source software that is freely accessible to all researchers. In addition to facilitating interactive visualization, the segmentations are anatomical representations that support derivation of morphological and mechanical measurements relevant to surgical planning. For example, previous work has demonstrated interactive manual measurement of geometric cusp height and commissural angle from segmentations (16), which are relevant to risk stratification for aortic valve repair. Future work will address automated computation of these and additional measurements. Volume rendering is an alternative technique for advanced visualization of volumetric data (6, 9). However, the quality of volume rendering can be variable and, unlike segmentation, volume rendering provides a visualization only and cannot inform automated quantification or assessment of mechanics (19).

Cases 7 through 9 illustrate how CT segmentation could potentially influence surgical planning, particularly the decision to repair or replace a regurgitant aortic valve. In Case 7, the CT segmentation revealed a TAV with large cusps and substantial calcification, rather than a possible BAV with mild calcification as interpreted on TTE. Although the trileaflet morphology and large cusps favored valve repair, the substantial calcification made surgical replacement more appropriate. Intraoperatively, the surgeon confirmed these findings and decided to replace rather than repair the valve. In Cases 8 and 9, the CT segmentations suggested more complex dysmorphisms than initially interpreted on TTE. Case 8 was variably interpreted on TTE as a TAV, BAV, or generally “abnormal,” but on CT segmentation appeared trileaflet with an accessory cusp containing a fistula. Case 9 was described as a “classic” Sievers Type 1 L/R fusion on TTE, but appeared to be consistent with a Sievers Type 2 BAV with a double raphe on CT segmentation. In both cases, the CT observations of complex dysmorphisms would shift the surgical plan to favor valve replacement over repair and thus influence the preoperative discussion between the surgeon and patient. It should be noted that while the CT findings in Case 7 were confirmed intraoperatively, the CT morphological interpretations of Cases 8 and 9 remain probable yet unverified, as intraoperative confirmation was not available.

This study has several limitations. First, the aortic valves were segmented manually, which is time consuming. Efforts are underway to automate this process (21) so that 3D aortic valve models can be generated rapidly for pre-operative planning. Secondly, given the small sample size of this exploratory study and the heterogeneity of the cohort and imaging protocols, it is not feasible to statistically report generalizable diagnostic accuracy. Future studies in larger populations are needed to validate the generalizability of these observations, characterize inter-observer variability in the visual interpretation of morphological features, and investigate the impact of 4D CT and PCCT diagnostics on surgical outcomes. Standardized segmentation protocols, automated quantification tools, and consensus reading frameworks could strengthen reproducibility and broaden clinical applicability of this work. Third, despite the value of contrast-CT in clarifying aortic valve morphology, it is not suitable for some patient populations, such as those who are pregnant, have renal dysfunction, or concerns related to radiation exposure. Retrospectively gated acquisitions performed without tube current modulation can be associated with much higher radiation doses than prospectively ECG-triggered exams, and PCCT imaging may increase dose further given reduced z-axis coverage and increased noise in the high-resolution mode. In this study, PCCT scans were performed at 120 kVp to allow spectral analysis, while EID-CT scans could be performed at lower kVp in patients with smaller body habitus. In Case 8 with smaller body habitus, the volume computed tomography dose index (CTDI_vol_) was 35.7 mGy with EID-CT and 79.9 mGy with PCCT. In Case 9 with larger body habitus, the CTDI_vol_ was 110 mGy with EID-CT and 68.5 mGy with PCCT. Thus in the smaller patient, lower dose was achieved on the EID-CT, but for the larger patient, the detector sensitivity allowed for lower dose with PCCT. With respect to limiting radiation exposure, high dose acquisition can be limited to the region of the aortic valve, and the remainder of the aorta imaged with low dose high-pitch helical technique. In addition, PCCT has been demonstrated to provide similar image noise at considerably reduced radiation dose compared to EID-CT, and developments in AI-based image reconstruction may allow dose to be reduced further. For this pilot study, we did not use ECG-gated tube current modulation; we are evaluating whether full dose imaging is needed throughout the cardiac cycle.

In summary, contrast-enhanced CT is routinely applied for the assessment of the aortic root and ascending aorta in adult BAV patients, and new techniques such as 4D acquisitions are already in use for planning transcatheter valve interventions. Further, PCCT is becoming more widely accessible, introducing the possibility of imaging at higher spatial resolution (slice thickness of 0.2 mm), as demonstrated in this study. Combining these advances with 3D image segmentation – which in the future may be automated with deep learning-based algorithms – provides an opportunity to visualize the aortic valve in its physiological, pressure-loaded state before the patient enters the operating room, which could benefit risk stratification and planning valve repair surgery, particularly in patients with unique morphology, as is often encountered in congenital aortic valve disease.

## Data Availability

The raw data supporting the conclusions of this article will be made available by the authors, without undue reservation.
